# The use of real-time PCR technique in the detection of novel protein 4.2 gene mutations that coexist with thalassaemia alpha in a single patient

**DOI:** 10.1111/j.1600-0609.2009.01289.x

**Published:** 2009-10

**Authors:** Monika Maciag, Anna Adamowicz-Salach, Alicja Siwicka, Justyna Spychalska, Beata Burzynska

**Affiliations:** 1 Institute of Biochemistry and BiophysicsPAS, Warsaw; 2 Department of Paediatrics, Haematology and Oncology, Medical University of WarsawWarsaw, Poland; 3 Institute of Haematology and Blood TransfusionWarsaw, Poland

**Keywords:** hereditary spherocytosis, protein 4.2 gene mutation, α-thalassaemia, PCR

## Abstract

α-Thalassaemia is a very rare disease in Northern Europe in contrast to hereditary spherocytosis that is associated with red blood cell membrane defects. We report here α-thalassaemia case who was also found to bear the erythrocyte membrane protein 4.2 gene mutations. mRNA relative quantification of red cell membrane protein genes in a Polish patient with α-thalassaemia trait indicated EPB42 as the gene that could also be involved in anaemia pathogenesis. Sequencing revealed the presence of two novel mutations in the protein 4.2 gene: a G1701A genetic change that predicts an alanine to threonine at position 567 of the protein (A567T) and a T→A substitution that is located at position +6 of the donor splice site of intron 2 (IVS2nt+6T>A). This is the sixth variant of the erythrocyte membrane protein 4.2 gene mutations identified outside the Japanese population.

Thalassaemia and hereditary spherocytosis (HS) are the most common inherited haemolytic anaemias, in general. However, α-thalassaemia is found mostly in patients of Mediterranean or South-East Asian origin and results from mutations affecting either one or both α-globin genes (α^+^-thalassaemia or α^0^-thalassaemia respectively). Subjects with three functional α-globin genes (−α/αα) are clinically and haematologically silent and those with two functional α-globin genes (−α/−α or –/αα, termed α-thalassaemia trait) present only very mild hypochromic microcytic anaemia (1).

In contrast, hereditary spherocytosis occurs in all racial groups and is particularly common in Northern European countries, with a prevalence of approximately 1 in 2000 (2).

The primary defects in HS reside in the erythrocyte membrane proteins that are involved in the interactions between the membrane skeleton and the lipid bilayer: spectrin, ankyrin, band 3 and protein 4.2 (3).

Isolated deficiency of protein 4.2 is a very common cause of HS in Japan (about 50% cases) but is rare in other populations, accounting for about 5% of all HS cases (4, 5). The only five protein 4.2 defects reported outside the Japanese population are: protein 4.2 Tozeur, Lisboa, Nippon, Nancy and Hammersmith, found in patients of Tunisian, Portuguese, Italian, French and South Asian origin respectively (6–10).

Here, we report the 3.7-kb α-thalassaemia-causing deletion, −α^3.7^/−α^3.7^ in a Polish patient who was also found to bear two novel protein 4.2 gene mutations discovered in the form of compound heterozygosity.

## Patient and methods

### Case report

Informed consent was obtained from the patient’s parent. The study protocol was approved by the Ethics Committee of the Medical University of Warsaw.

The analysed patient has been under the care of the outpatient department of haematology since birth because of haemolytic anaemia of unknown aetiology. The girl was initially treated with ferrum supplements, but without effect. She had never undergone transfusion and had never been treated with hematopoietic drugs. The RBC parameters did not clearly indicate the cause of the anaemia (Table 1). Eosin-5-maleimide (EMA) labelling of red blood cells and flow cytometry were performed as a screening test for HS and did not reveal a decrease in fluorescence intensity. Sodium dodecyl sulphate-polyacrylamide gel electrophoresis (SDS-PAGE) results did not shown prominent changes in erythrocyte membrane protein levels. Therefore, a diagnosis of hereditary spherocytosis was initially excluded. Enzymopathy of red cells (glucose-6-phosphate dehydrogenase – 18.3, hexokinase – 1.3, glucose phosphate isomerase – 36.7, pyruvate kinase – 14.3 jm/gHb) and autoimmunohaemolytic anaemia were also excluded.

**Table 1:** Haematological parameters of the proband

	Age	Sex	RBC (10^6^/μL)	Ret (%)	Ht (%)	Hb (g/dL)	MCV (fL)	RDW (%)	MCH (pg)	MCHC (g/dL)	HbA_2_ (%)	HbF (%)
Normal value	–	F	4.2–5.4	0.6–2.6	37–47	12–16	81–99	11–15	27–31	32–36	>2.5	>1
Proband	16	F	4.67	6.6	28.7	8.7	61.4	24.1	18.7	30.4	2.0	1.1

Ret, reticulocytes; Ht, hematocrit; MCHC, mean corpuscular haemoglobin concentration; HbA2, haemoglobin A2; HbF, haemoglobin F; MCV, mean corpuscular volume; RDW, red cell distribution width; MCH, Mean corpuscular volume.

**Figure 1:**
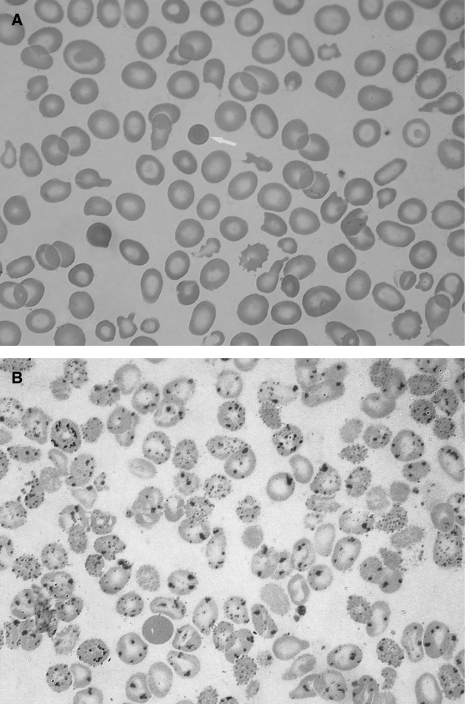
(A) May–Grunwald–Giemza staining of erythrocytes (microspherocyte marked with arrow). (B) Reticulocytes staining performed with brilliant cresyl blue.

The blood smear showed microcythosis (Fig. 1). The bilirubin level ranged from 1.5 to 1.8 mg/dL (max). Additional studies revealed a ferritin level between 70 and 180 ng/mL, lactate dehydrogenase 448–662 U/L and Fe 47.0–105 μg/dL. Abdominal ultrasonography examination showed splenomegaly (138 mm).

Blood studies of the patient’s mother (conducted outside of outpatient clinic) suggested a thalassaemic trait: decreased levels of mean corpuscular volume (MCV – 69.7 fL), mean corpuscular haemoglobin (MCH – 21.9 pg), and an RBC count of 5.52 (10^6^/μL). The patient’s brother and sister were not anaemic and her father was not available to the study.

### Quantitation of erythrocyte membrane proteins

A flow cytometric test with the use of eosin-5-maleimide, a dye that covalently reacts with Lys430 of the band 3 protein (11), was performed to detect membrane-associated inherited haemolytic anaemia. Erythrocyte membrane protein levels were measured by SDS-PAGE.

### RNA isolation and reverse transcription

Total RNA was isolated from peripheral blood samples using the PAXgene Blood RNA Kit (QIAGEN, Hilden, Germany). Reverse transcription was performed using QuantiTect Reverse Transcription Kit (QIAGEN) according to the manufacturer’s recommendations.

### mRNA relative quantification

mRNA relative quantification for the genes of erythrocyte membrane proteins was carried out using real-time polymerase chain reaction (PCR). qPCR amplification was performed using LightCycler 1.5 and LightCycler FastStart DNA Master SYBR Green I (Roche Diagnostics GmbH, Mannheim, Germany) according to the manufacturer’s recommendations. The Pfaffl model (12) and the relative expression software tool (rest-384^©^) (13) were used to estimate the relative changes in mRNA levels of ankyrin, spectrins, band 3 and protein 4.2 as well as globin genes in the analysed patient (compared with 10 healthy individuals). Data normalisation was carried out against the transcript of the gene for EMP55 (erythrocyte membrane protein p55).

### DNA isolation

A DNA Isolation Kit for Blood/Bone Marrow/Tissue (Roche Diagnostics GmbH) was used to isolate DNA from blood samples.

### Detection of the α-thalassaemia deletion −α^3.7^ by multiplex PCR

The multiplex PCR method was utilised for detection of the common α-thalassaemia deletion −α^3.7^, as previously described (14).

### Detection of protein 4.2 gene mutations

PCR was used to amplify the entire coding sequence, promoter region and surrounding sequences of the EPB42 gene. DNA fragments generated by PCR amplification were purified using QIAquick PCR Purification Kit (QIAGEN) and directly sequenced. As the detected nucleotide substitution creates a new restriction site, we performed restriction analysis with the RsaI enzyme (Roche Diagnostics GmbH) to confirm the presence of the A567T mutation. The IVS2nt+6T>A substitution was verified by sequencing of a separate PCR product, obtained with a separate set of primers, different from those used for the initial sequencing. The absence of the identified genetic changes was confirmed among at least 50 healthy individuals. The sequences of all primers and the annealing temperatures used in this study are available upon request.

## Results

### Quantitation of erythrocyte membrane proteins

EMA labelling of red blood cells and flow cytometry did not reveal a decrease in fluorescence intensity in the proband (92.99% of control). The erythrocyte membrane protein levels of the analysed patient were normal (data not shown). Her family members were not available for the molecular studies.

### Globin genes mRNA relative quantification and identification of the α-thalassaemia deletion −α^3.7^

mRNA relative quantification of the globin genes revealed a decreased α-globin transcript level when compared with the control group (Table 2). DNA analysis of the α-globin gene showed the presence of the 3.7 kb deletion within the α-globin gene cluster (−α^3.7^/−α^3.7^) in the analysed patient.

**Table 2:** Relative mRNA levels of the globin and erythrocyte membrane protein genes in the analysed patient

	α-Globin	β-Globin	ANK1	SLC4A1	SPTA1	SPTB	EPB42
Proband	0.27 (0.001)	2.09 (0.002)	2.08 (0.001)	2.45 (0.003)	2.18 (0.007)	0.79 (0.326)	0.55 (0.032)

*P*-values are given in brackets.

ANK1, ankyrin, erythrocytic; SLC4A1, solute carrier family 4, anion exchanger, member 1, band 3; SPTA1, spectrin, alpha, erythrocytic 1; SPTB, spectrin, beta, erythrocytic; EPB42, protein 4.2, erythrocytic.

### Erythrocyte membrane protein genes mRNA relative quantification and the identification of mutations in the protein 4.2 gene

**Figure 2:**
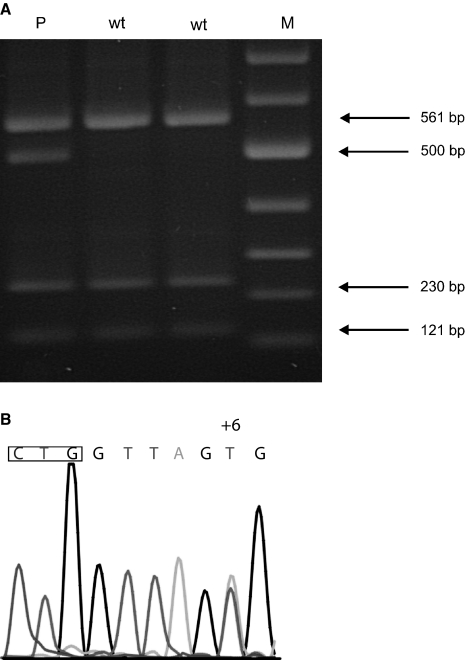
(A) Pattern of RsaI digestion of the PCR product containing exon 10 (detection of G1701A substitution in the EPB42 gene with genomic DNA as a template): M, 100 bp marker; P, proband; wt, wild-type. (B) Sequence analysis of the fragment around the donor splice site of intron 2 of the EPB42 gene (the three last nucleotides of the EPB42 exon 2 were put in rectangle; genomic DNA was used as a template).

Relative mRNA quantification was carried out for the red blood cell membrane protein genes at the same time. The patient displayed increased transcript levels of the genes for ankyrin, band 3 and spectrin α, and an approximately 45% decrease in the relative mRNA level of the gene encoding the protein 4.2 (EPB42) in comparison with the control group (Table 2). The next stage of our studies involved sequencing of the protein 4.2 gene to find a cause of the decreased mRNA level. Direct sequencing of the EPB42 gene revealed the presence of two previously undescribed mutations in heterozygotic form in the proband. The G1701A mutation is located in exon 10 and predicts an alanine to threonine change at position 567 (A567T). The presence of this substitution at the DNA level was verified using RsaI restriction enzyme analysis (Fig. 2A) and by sequencing at the cDNA level. The second genetic change identified, the T→A substitution, is located at position +6 of the donor splice site of intron 2 (IVS2nt+6T>A). The presence of this mutation was confirmed by sequencing (Fig. 2B).

## Discussion

The case of the hereditary haemolytic anaemia described here represents an atypical type of α-thalassaemia that coexists with the erythrocyte membrane protein 4.2 gene mutations. Protein 4.2 defects are known to cause hereditary spherocytosis. However, α-thalassaemia and HS are rarely inherited together. Few reports (15, 16) are known to describe combinations of hereditary spherocytosis and α-thalassaemia. Heaton *et al.* 1991 (15) conclude that patients with both diseases had milder presence of spherocytosis. Li *et al.* 1994 (16) state that the haemolytic effect of hereditary spherocytosis is modulated by the increased osmotic resistance of red blood cells in patients with both disorders. Similar results were shown by del Giudice *et al.*, 1993 (17) who demonstrated that β-thalassaemia traits ‘silence’ most of the clinical findings associated with HS.

In our patient, the coexistence of the erythrocyte membrane protein 4.2 gene mutations and α-thalassaemia causing deletions results in a decrease in haemoglobin concentration, microcytosis, and also an increasing red cell distribution width (RDW) value.

A possible direct interaction between the globin chain and membrane proteins could have had an effect on the sensitivity of the EMA test. Due to a lack of clear conclusions from the SDS-PAGE and EMA tests, mRNA relative quantification of the globin and red blood cell membrane protein genes was introduced for further investigation of the cause of anaemia in the proband.

The decreased level of the α-globin transcript in the analysed patient prompted us to perform DNA analysis of the alpha globin gene. Thus, the 3.7 kb deletion within the α-globin gene cluster (−α^3.7^/−α^3.7^) was detected in the proband.

In addition, mRNA relative quantification of the red cell membrane protein genes in the Polish patient indicated the EPB42 gene as the one that could also be involved in anaemia pathogenesis. Sequencing analysis revealed the presence of two novel mutations in the protein 4.2 gene: a G1701A genetic change that predicts an alanine to threonine at position 567 of the protein (A567T) and a T→A substitution that is located at position +6 of the donor splice site of intron 2 (IVS2nt+6T>A). Because of the location near the 5′ splice site of the intron 2, the T→A substitution in the sixth nucleotide of intron 2 of the EPB42 gene could reduce activity of this splice site and lead to mRNA instability. It has already been shown that mutations at position +6 of the β-globin gene intron affect its splicing (18) and this result was recently confirmed by our own work, in which we show marked reduction in the relative amount of β-globin transcript in patients carrying genetic changes at position +6 of the β-globin gene intron (19). However, the T→A substitution in the sixth nucleotide of intron 2 of the EPB42 gene detected in Polish patient does not totally destabilise mRNA carrying this genetic change. This was confirmed by sequencing cDNA product across the A567T substitution that revealed heterozygotic form of the A567T mutation (data not shown). The nucleotide substitution IVS2nt+6T>A is the second splicing affecting mutation reported in the EPB42 gene so far. The previous one, called protein 4.2 Notame, was found in a Japanese man and involved a single nucleotide substitution G→A in the first nucleotide of intron 6 (20). The vast majority of genetic changes identified in the EPB42 gene are missense mutations (21) and most of them were found in Japan. The only five protein 4.2 variants identified outside the Japanese population are: protein 4.2 Tozeur (R310Q) (6), protein 4.2 Lisboa (136/137 delG) (7), protein 4.2 Nippon (T142A) (8), protein 4.2 Nancy (949 delG) (9) and protein 4.2 Hammersmith (1747G>T) (10). Our patient, who was found to be a compound heterozygote for two novel mutations in the EPB42 gene, represents the fourth case of the EPB42 gene mutations discovered in the European population.

In conclusion, our data suggest that measurement of mRNA levels of globin and erythrocyte membrane protein genes by real-time PCR can be useful for genetic screening of patients with not clarified hereditary haemoytic anaemia, especially for cases with down-regulated gene expression or mRNA instability.
